# Aqueous Extract of *Mori Folium* Exerts Bone Protective Effect Through Regulation of Calcium and Redox Homeostasis via PTH/VDR/CaBP and AGEs/RAGE/Nox4/NF-κB Signaling in Diabetic Rats

**DOI:** 10.3389/fphar.2018.01239

**Published:** 2018-11-06

**Authors:** Chenyue Liu, Ruyuan Zhu, Haixia Liu, Lin Li, Beibei Chen, Qiangqiang Jia, Lili Wang, Rufeng Ma, Simin Tian, Min Wang, Min Fu, Jianzhao Niu, Alexander N. Orekhov, Sihua Gao, Dongwei Zhang, Baosheng Zhao

**Affiliations:** ^1^School of Chinese Material Medica, Beijing University of Chinese Medicine, Beijing, China; ^2^Diabetes Research Center, Traditional Chinese Medicine School, Beijing University of Chinese Medicine, Beijing, China; ^3^Guang'anmen Hospital, China Academy of Chinese Medical Sciences, Beijing, China; ^4^The Research Institute of McGill University Health Center, Montreal, QC, Canada; ^5^Laboratory of Angiopathology, Institute of General Pathology and Pathophysiology, Moscow, Russia; ^6^Beijing Research Institute of Chinese medicine, Beijing University of Chinese Medicine, Beijing, China

**Keywords:** *Mori Folium*, bone quality, diabetes, calcium homeostasis, redox equilibrium, PTH/VDR/CaBP, AGEs/RAGE/Nox4/NF-κB

## Abstract

**Purpose:** The present study is aimed to explore whether the aqueous extract of *Mori Folium* (MF) exhibits bone protective effect by regulating calcium and redox homeostasis in diabetic rats, and to identify the signaling pathways involved in this process.

**Methods:** Diabetic rats were established using high-sugar and high-fat diet and streptozotocin (STZ) (30 mg/kg for 3 consecutive days). The serum levels of osteocalcin (OC), insulin-like growth factor-1 (IGF-1), tartrate-resistant acid phosphatase (TRAP), phosphorus (P), calcium (Ca), 1,25-dihydroxyvitamin D_3_ [1,25(OH)_2_D_3_], parathormone (PTH), advanced glycation end products (AGEs), superoxide dismutase (SOD), and malondialdehyde (MDA), total antioxidant capacity (TAC), 8-hydroxy-2′-deoxyguanosine (8-OH-dG), and interleukin 6 (IL-6) were determined by ELISA or biochemical assays. Histopathological alterations in the femurs were evaluated by the stainings of hematoxylin-eosin (H&E) and alizarin red S. In addition, femoral strength was detected by a three-point bending assay, bone microstructure was detected with micro-computer tomography. Bone material properties were examined by Fourier-transform infrared spectroscopy. Furthermore, the expressions of IGF-1, runt-related transcription factor 2 (Runx2), osteoprotegerin (OPG), receptor activator of nuclear factor kappa-B ligand (RANKL), cathepsin K, AGEs, receptor of advanced glycation end products (RAGE), NADPH oxidase 4 (Nox4), and nuclear factor kappa-B (NF-κB) in the femurs and tibias, and the alterations in the levels of calcium-binding protein-28k (CaBP-28k), transient receptor potential V6 (TRPV6), and vitamin D receptor (VDR) in the kidneys and duodenums were determined by western blot and immunohistochemical analysis.

**Results:** Treatment of diabetic rats with MF aqueous extract induces an increase in the levels of OC and IGF-1 as well as a decrease in TRAP level in serum. MF treatment also upregulates the expression of OPG, downregulates the expressions of AGEs, RAGE, Nox4, NF-κB, and RANKL, which leads to improve bone microstructure and strength exhibited by an increase in cortical area ratio, cortical thickness, and trabecular area ratio as well as ultimate load, elastic modulus, and bending stress in the femurs and tibias of diabetic rats. In addition, MF aqueous extract preserves bone material properties by decreasing the ratio of fatty acid/collagen and increasing the ratio of mineral/matrix in the femurs of diabetic rats. Moreover, MF treatment increases the levels of P, Ca, and 1,25(OH)_2_D_3_, and decreases the level of PTH in the serum, as well as upregulates the expressions of TRPV6 and VDR in the duodenums and CaBP-28k in the kidneys of diabetic rats. Additionally, MF has ability of rebuilding redox homeostasis and eliminating inflammatory stress by increasing the levels of SOD and TAC as well as decreasing the levels of IL-6, AGEs, MDA, and 8-OH-dG.

**Conclusions:** MF treatment may improve bone quality through maintenance of calcium homeostasis via regulating the PTH/VDR/CaBP signaling, and elimination of oxidative stress via regulating the AGEs/RAGE/Nox4/NF-κB signaling. These results may suggest the potential of MF in preventing the development of diabetic osteoporosis.

## Introduction

*Mori Folium* (MF), known as Sangye (Pinyin name) in Chinese, is the dried leaf derived from *Morus alba* L., which has been widely used for more than 1000 years in Chinese medicine clinics and other Asian countries (Tian et al., 2016). According to Chinese Pharmacopeia (2015 version), MF is yearly harvested at the First Frost and recognized as an edible herb with the function of clearing heat and eliminating cough as well as invigorating liver and improving eyesight in combination with other herbs. Phytochemical studies reveal that MF contains at least 11 compounds, such as isochlorogenic acid, 5,7- dlhydroxycommarin-7-O-β-D-glucopyranoside, scopolin, chlorogenic acid, kaempferol-3,7-dl-O-β-D-glucopyranoside, 4-caffeoylquinic acid methyl ester, rutin, hyperoside, isoquercitrin, astragalin, isochlorogenic acid (Tang et al., 2016) (Supplementary Figure 1), which exhibits a wide spectrum of biological activities, including regulation of blood glucose (Jang et al., 2002; Cai et al., 2016) and lipids (Jang et al., 2002; Kim et al., 2015) metabolism, and elimination of inflammation and oxidative stress (Jeong et al., 2017; He et al., 2018).

Characterized by higher risk of bone fracture, reduction of bone strength, and deterioration of bone microarchitecture, diabetic osteoporosis has been recognized as one of severe complications during the development of diabetes (Ma et al., 2017). Its incidence is yearly increased with an alarming rise in the population of diabetic patients (Ma et al., 2016). Hyperglycemia negatively affects bone formation by inhibition of calcium absorption and reabsorption in the duodenum and kidneys through regulation of transcellular calcium transporting proteins via decreasing the expressions of vitamin D receptor (VDR), transient receptor potential V6 (TRPV6), and calcium-binding protein-28k (CaBP-28k) (Zhang et al., 2011, 2014). In addition, hyperglycemia leads to a negative calcium balance and subsequent bone demineralization through regulation of the calcium–parathyroid hormone (PTH) axis (Napoli et al., 2017). Furthermore, PTH is demonstrated to be negatively correlated with bone mineral density (BMD) and positively correlated with osteoporotic fracture in the patients with type 2 diabetes (Wang et al., 2018). Sustained exposure to high glucose may impair bone formation through reducing 1,25-dihydroxyvitamin D_3_ [1,25(OH)_2_D_3_] receptor numbers (Inaba et al., 1995). And supplement of 1,25(OH)_2_D_3_ may inhibit the progress of diabetic associated complications (Ding et al., 2016; Wang et al., 2016).

Chronic hyperglycemia triggers oxidative stress and overproduction of advanced glycation end products (AGEs) (Napoli et al., 2017). The interaction of AGEs with receptor of advanced glycation end products (RAGE) further promotes reactive oxygen species (ROS) generation and nuclear factor kappa-B (NF-κB) activation through AGE-RAGE-NADPH oxidase (Nox) signaling (Tóbon-Velasco et al., 2014; Hou et al., 2017), which followed by an increase in osteoclastogenesis and subsequent bone resorption (Wang et al., 2017). Interestingly, our previous results demonstrated that the activation of Nox4/ROS/NF-κB signaling could lead to bone loss and the progression of osteoporosis (Wang et al., 2017). In addition, the accumulation of AGEs damages bone quality through interfering with bone material constituents and biomechanical properties via inhibition of mature nodule formation and mineralization of osteoblasts (Napoli et al., 2017). Interestingly, emerging evidences suggest that natural products show promising effect on alleviating multifactorial metabolic aspects of osteoporosis by its anti-oxidant and anti-inflammatory activities (Tabatabaei-Malazy et al., 2015; Menghini et al., 2016).

Recent studies from our lab and other investigators have demonstrated that MF has the ability of preventing the development of diabetes and its associated disorders by inhibiting nitric oxide synthase expression (Jang et al., 2002), increasing neuropeptide Y expression in the dentate gyrus (Kim et al., 2003), eliminating oxidant stress (Huang et al., 2014; Kim et al., 2014), and regulating insulin receptor substrate 1/phosphoinositide 3-kinase/glucose transporter type 4 signaling (Cai et al., 2016) in diabetic rats. Furthermore, MF water extract has been reported to alleviate articular cartilage damages and inhibit inflammatory responses in monosodium iodoacetate-induced osteoarthritis rats (Jeong et al., 2017). The combined extracts of *M. alba* and *Polygonum odoratum* exhibited bone protective effect through decreasing osteoclastogenesis and increasing osteoblastogenesis and cortical thickness via reducing oxidative stress and elevating serum calcium and osteocalcin (OC) in ovariectomized (OVX) rats (Sungkamanee et al., 2014). However, whether MF exhibits anti-osteoporotic activities in the development of diabetes and its underlying mechanisms still remain unclear. To this end, in the current study we hypothesized that MF may improve bone quality through its regulation of PTH/VDR/CaBP and AGEs/RAGE/Nox4/NF-κB signaling in diabetic rats.

## Materials and methods

### Chemicals and antibodies

Alizarin red S were purchased from Sigma-Aldrich (St. Louis, MO, USA). Antigen retrieval solution was from ShunBai Biotechnology Company (No: SBT10013; Shanghai, China). Calcium (Ca), phosphorus (P), SOD, malondialdehyde (MDA), total antioxidant capacity (TAC) kits were purchased from Nanjing Jiancheng Bioengineering Institute (Nanjing, China). OC, IGF-1, TRAP, 1,25(OH)_2_D_3_, PTH, AGEs, and 8-OH-dG ELISA kits were purchased from Beijing Fangcheng Biotechnology Company (Beijing, China). Interleukin 6 (IL-6) and tumor necrosis factor alpha (TNF-α) ELISA kits were obtained from Thermo Fisher Company (Massachusetts, USA). Antibodies against runt-related transcription factor 2 (Runx2) (ab76956), cathepsin K (ab19027), OPG (ab73400), RANKL (ab62516), AGEs (ab23722), RAGE (ab3611), Nox4 (ab133303), NF-κBp65 (ab16502) were purchased from Abcam Biocompany (Cambridge, MA, USA). Antibodies against IGF-1(H70) (sc9013), VDR (D-6) (sc-13133), and Calbindin D28k (CaBP-28k) (D-4) (sc-365360) were from Santa Cruz Biotechnology (Dallas, TX, USA). Anti-TRPV6 polyclonal antibody (Cat. No: 13411-1-AP) was from Proteintech (Rosemont, USA).

### Preparation of MF aqueous extract

MF aqueous extract was prepared as previously described (Tang et al., 2016). Briefly, *Mori Folium* (MF) was purchased from Beijing TongRenTang pharmacy (Beijing, China) and authenticated by Professor Guijun Zhang [Chinese Material Medica School, Beijing University of Chinese Medicine (BUCM)]. Then, 300 g of grinded raw MF (Mulberry leaves) was soaked in 3,000 ml distilled water for 10 h at 85°C for twice, and followed by filtering and concentrating the supernatants to 0.2 g/ml under vacuum.

### Animal model establishment

Sixty 3-month-old male Wistar rats, weighing about 220 ± 20 g, were purchased from Beijing Sibeifu Animal Technology Co. Ltd. [Beijing, China; certification No: SCXK (Jing) 2016-0002]. The rats were housed in the SPF clean animal housing facilities [certification number SYXK (Jing) 2016-0038] of BUCM, temperature of 22 ± 1°C, humidity of 55 ± 5%, and a 12-h light/dark cycle with free access to tap water and chow. All the protocols were reviewed and approved by the Animal Care Committee of BUCM, China.

After acclimation for 1 week, 12 rats were randomly selected into normal control (NC) group and fed with a regular chow. The rest of rats were fed with a high-sugar and high-fat diet (HSD-HFD), which was provided by Beijing Ke'ao Xieli Food Co. Ltd. (Beijing, China). Ten weeks later, the rats were intraperitoneally injected with freshly prepared STZ [30 mg/kg dissolved in 0.1 mM citrate buffer (pH4.2)] or vehicle [0.1 mM citrate buffer (pH4.2)] daily for 3 consecutive days. Three days after the induction of diabetes, the rats whose random blood glucose level was higher than 12 mM were collected as diabetic rats and randomly divided into four groups, namely, diabetes mellitus model control group (DM), metformin-treated control group (Met), MF high dose-treated group (MFH), and MF low dose-treated group (MFL). The rats in Met group were daily administrated with Met at a dosage of 500 mg/kg/d by gavage. The rats in MFH and MFL group were daily gavaged with 2 and 1 g/kg/d of MF aqueous extract, respectively. The rats in DM, Met, MFH, and MFL were fed on the HSD-HFD until the end of the study. The rats in NC and DM groups were orally administrated with an equal volume of distilled water. Fasting blood glucose (FBG) levels were monitored by blood glucometer (Johnson; New Brunswick, NJ, USA) every 4 weeks. All the rats were treated for 12 weeks.

### Oral glucose tolerance test (OGTT)

For OGTT, the rats were fasted overnight, then FBG levels from the tail vein were determined at 0, 30, 60, 90, and 120 min after oral administration of glucose (2 g/kg). The glucose tolerance was evaluated by calculating the area under the curve (AUC). AUC was calculated as following: AUC(mmol·h/L) = 0.25 × BG_0_ + 0.5 × B G_30_ + 0.75 × BG_60_ + BG_120_.

### Serum biomarkers determination

After 12 weeks of intervention, all rats were euthanized with 1% sodium pentobarbital (0.4 ml/100 g, i.p.). Blood samples were collected from abdominal aorta and stored for biochemical assay. Serum levels of Ca, P, SOD, MDA, and TAC were measured by commercial biochemical kits using a microplate reader (FLUO star Omega; Ortenberg, Germany). And the serum levels of OC, IGF-1, TRAP, 1,25(OH)_2_D_3_, PTH, AGEs, IL-6, TNF-α, and 8-OH-dG were determined by ELISA.

### The stainings of H&E and alizarin red S

The left femurs of the rats were first immersed in 10% neutral formalin for 72 h, and then decalcified in 15% neutral EDTA buffer for 3 months. The decalcified femurs were further dehydrated and defatted with graded ethanol (50–100%) and xylene, and embedded in paraffin. Sections of ~4-μm thickness were used for the H&E staining according to the routine protocol (Guo et al., 2016).

Alizarin red S staining was performed according to the protocol as previously published (Guo et al., 2016). Briefly, the dehydrated slides were stained with alizarin red S solution for 2 min, followed by quickly dipped into acetone and acetone xylene (50/50), each for 20 s. Then the slides were dehydrated with graded alcohol and cleared in xylene.

After staining, the mounted slides were observed and photographed using an Olympus BX53 fluorescence microscope (Tokyo, Japan). The tissue sections were examined for histopathological changes, including the structure and morphology of trabecular bone. The relative interest of density values of alizarin red S staining was quantified using the Image Pro Plus 6.0 software.

### Micro-computed tomography (μCT) scanning

The right femurs of the rats which were cleaned of adhering soft tissues were placed in the 48-mm specimen holder, and followed by scanning with a μCT apparatus for small experimental animals (Model LaTheta LCT-200; Hitachi-Aloka, Tokyo, Japan), which was operated at 50 kV and 0.5 mA (radiation exposure remained below 40 mSv) for measurements (Ueyama et al., 2013). Briefly, an overview scan of the whole bone was firstly created to allow the selection of regions of interest for the different scans. Then, the areas between the proximal and the distal end of femur were scanned to quantify cortical and spongy bones. For all scans, the same number of views was used, which represents the number of data collected during a single 360° -rotation around the object. The parameters, including cortical bone thickness, cortical bone area ratio, and trabecular bone area ratio were calculated automatically by the LaTheta software (version 3.20).

### Bone biomechanical strength assay

After μCT scanning, the right femurs were taken for a three-point bending assay by electronic universal testing machine (Shenzhen Reger Instrument Co. Ltd., Model RGWF4005, China). The shaft of the femur was fixed between two supporting points, with a distance of 20 mm. A load was vertically applied to the femoral midshaft, with a displacement speed of 0.01 mm/s at 100 Hz until the femoral shaft is fractured. The ultimate load, bending strength, and elastic modulus of the femurs were recorded.

### FTIR spectrum data acquisition

The rat femurs were grinded to powders with liquid nitrogen in a ceramic mortar. Then 1% (wt/wt) of femoral powders were mixed with KBr during grinding and pressed into an optical transparency pellet (10 tons, 30 s). In total, 45 separated KBr tablets were prepared, scanned, and analyzed. FTIR spectrum were acquired by FTIR spectrometer (PerkinEImer, America). Scanning was performed in transmission mode in the 4,000–400 cm^−1^ range at 4 cm^−1^ resolution with accumulating 64 scans (Tang et al., 2016). Finally, fatty acid/collagen was determined by the areas of fatty acid (~1,720–1,780 cm^−1^) divided by the areas of amide I band (~1,592–17,28 cm^−1^). Collagen maturity (also referred to as collagen cross-link ratio) was calculated from the relative intensity ratios of the 1,660 to 1,690 cm^−1^ peaks (Farlay et al., 2011). The relative mineralization of the collagen matrix (mineral/matrix) was determined by the areas of phosphate r1, v3 band (~900–1,159 cm^−1^) divided by the areas of amide I band (~1,592–1,728 cm^−1^) (Paschalis et al., 2011).

### Immunohistochemical (IHC) staining

IHC staining was conducted according to the procedure as previously described (Ma et al., 2017) with some modifications. Briefly, 4 μm longitudinal sections of the paraffin embedded femurs were kept in the oven at 60°C for 24 h and then followed by defatting with xylene and hydrating with graded ethanol (100–70%). Then slides were sequentially incubated with an antigen retrieval solution (Shanghai ShunBai Biotechnology Company, China), 3% H_2_O_2_ for 30 min, and incubated with a primary antibody [IGF-1(1:50), VDR (1:50), OPG (1:50), RANKL (1:100), CaBP-28k (1:50) or AGEs (1:300)] overnight at 4°C. For negative controls, the primary antibodies were replaced by non-immunized goat serum. Eighteen hours later, the slides for IHC staining were incubated with corresponding secondary antibodies (Beijing Biosynthesis Biotechnology Co. Ltd., China) for 10 min followed by DAB and hematoxylin staining. Finally, the slides of IHC staining were examined and photographed using Olympus BX53 microscope. The intensity of DAB staining was analyzed using Image Pro Plus 6.0 software and expressed as IOD value.

### Western blot assay

The tibias, kidneys, and duodenums were homogenized and extracted in RIPA buffer supplemented with complete protease inhibitor cocktail (Beyotime Biotechnology, Shanghai, China), and the protein concentration was determined using a BCA protein assay kit (Thermo Scientific, USA). A total of 50 or 100 μg of denatured protein samples from the tibias or kidneys or duodenums were separated by 10% SDS-PAGE gel and then transferred onto a PVDF membrane. After being blocked with 5% skim milk in TBST (Tris-buffered saline containing 0.1% Tween 20) for 1 h at room temperature, the membranes were then incubated overnight at 4°C with appropriate primary antibodies [Runx2 (1:1,000), cathepsin K (1:1,000), VDR (1:500), TRPV6 (1:1,000), CaBP-28k (1:250), AGEs (1:1,000), RAGE (1:1,000), Nox4 (1:1,000), NF-κB (1:1,000)] followed by incubation with the corresponding HRP-labeled secondary antibody (1:1,000 or 1:2,000) at room temperature for 2 h on the second day. Immuno-positive bands were visualized with high sensitivity ECL luminous liquid and the images were captured with Azure Bio-imaging systems (California, USA). The gray values of the bands were quantified using the Image J software, and normalized with the corresponding β-actin (1:5,000) as the internal control.

### Statistical analysis

All the data obtained from animal experiments of different groups were quantitative data. When the data meets a normal distribution and variances are homogeneous, One-way analysis of variance (ANOVA) test is used to analyze data. When the data meets a normal distribution but the variances are not homogeneous, Dunnett's T3 test is used to analyze data. When the data not meet a normal distribution, nonparametric analysis is used. Statistical significance was determined at *p* < 0.05.

## Results

### MF decreased FBG and AUC of OGTT in diabetic rats

**Figure 1:**
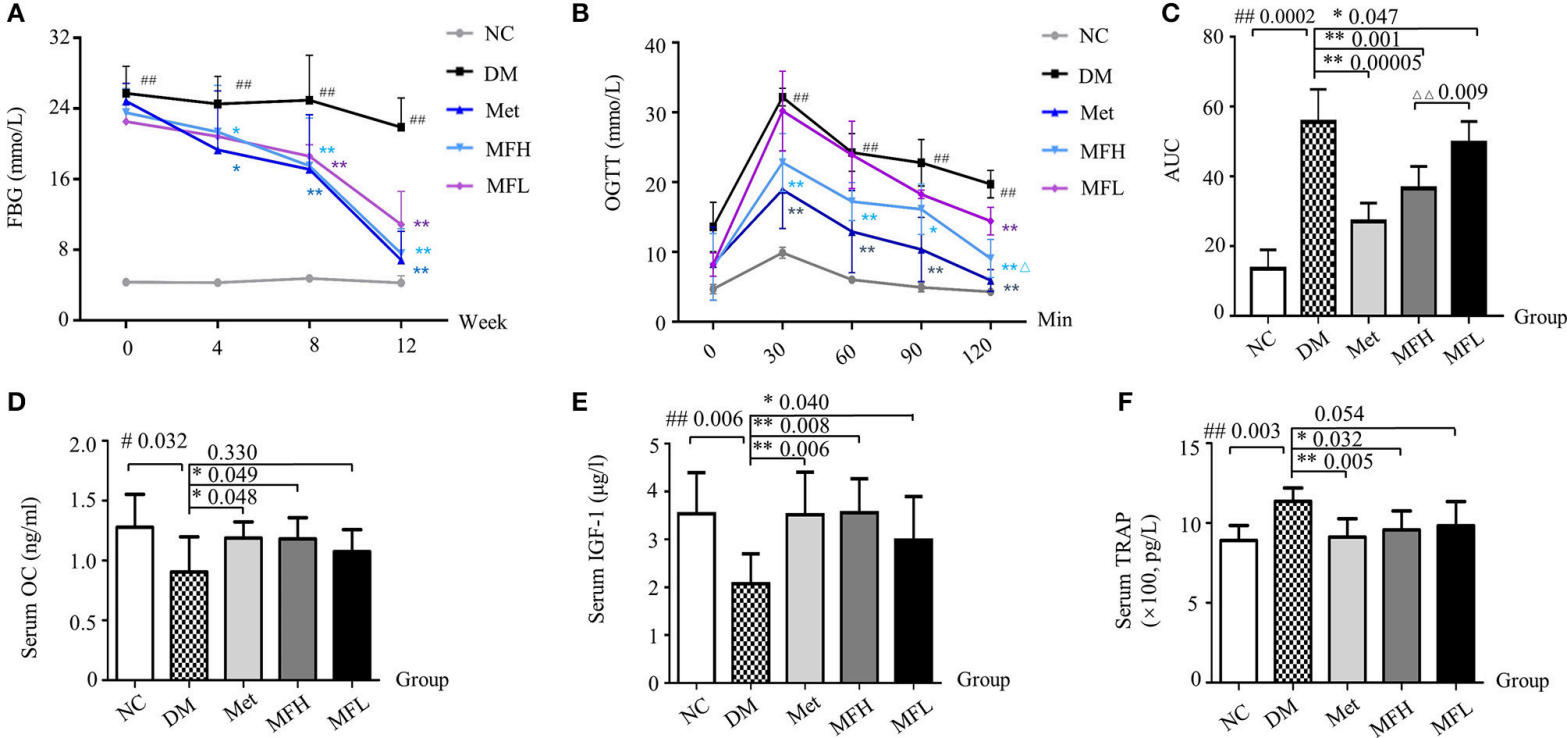
MF reversed serum markers of blood glucose and bone formation in diabetic rats. **(A)** Shows the fasting blood glucose levels in the different group of rats at 0, 4, 8, 12 weeks. **(B,C)** Show OGTT and AUC in the different groups of rats at 0, 30, 60, 90, 120 min. **(D–F)** Show the results of serum levels of OC, IGF-1, and TRAP in the different group of rats. Data are presented as mean ± SD. *n* = 12. ^#^*p* < 0.05 or ^##^*p* < 0.01 compared with NC group, *p < 0.05 or **p < 0.01 compared with DM group, ^ΔΔ^*p* < 0.01 compared with MFL group.

As expected, the FBG levels in diabetic rats were significantly higher than in the NC rats. From 4 weeks on, the FBG levels in the rats of MFH and Met group were lower than those in DM group (*p* < 0.01). And from 8 weeks on, the FBG levels of the diabetic rats in MFL group were significantly reduced (*p* < 0.01), as compared with those of the rats in DM group (Figure 1A).

At the end of the intervention, OGTT was determined. As shown in Figures 1B,C, the AUC of OGTT in the rats of DM group were significantly increased when compared with that in NC group (*p* < 0.01). Interestingly, the AUC of OGTT in the rats of MFH and MFL and Met groups were significantly lower than those in DM group (*p* < 0.05 or 0.01). And MFH show better capacity in controlling blood glucose than MFL (*p* < 0.01). These results suggest that MF aqueous extract has the ability of improving glucose tolerance in diabetic rats.

### MF increased serum levels of bone formation markers and decreased levels of bone resorption markers

Increased OC level helps to improve glucose homeostasis in diabetes (Kanazawa, 2015). And the increased TRAP expression in diabetic rats reflects a trend toward bone loss (Rivoira et al., 2018). Meanwhile, the increased IGF-1 level in serum may reduce the risk of non-vertebral fractures in patients with type 2 diabetes (Miyake et al., 2017). Indeed, as shown in Figures 1D,E, the serum levels of OC and IGF-1 were significantly decreased in the rats of DM group as compared to those in NC control (*p* < 0.01 or 0.05). As expected, both Met and MF (MFH and MFL) treatment significantly prevented the reduction of serum OC and IGF-1 levels compared to vehicle treatment in diabetic rats (*p* < 0.01 or 0.05). In addition, the serum TRAP level in the vehicle treated diabetic rats were higher than in NC rats (*p* < 0.05). Treatment of diabetic rats with Met and MF aqueous extract significantly reduced the serum TRAP level compared with that of vehicle treated diabetic rats (*p* < 0.05, Figure 1F). And MFH is better in promoting bone formation than MFL. These findings indicate that MF may have the ability of rebuilding bone homeostasis.

### MF improved femoral histopathological alterations in diabetic rats

**Figure 2:**
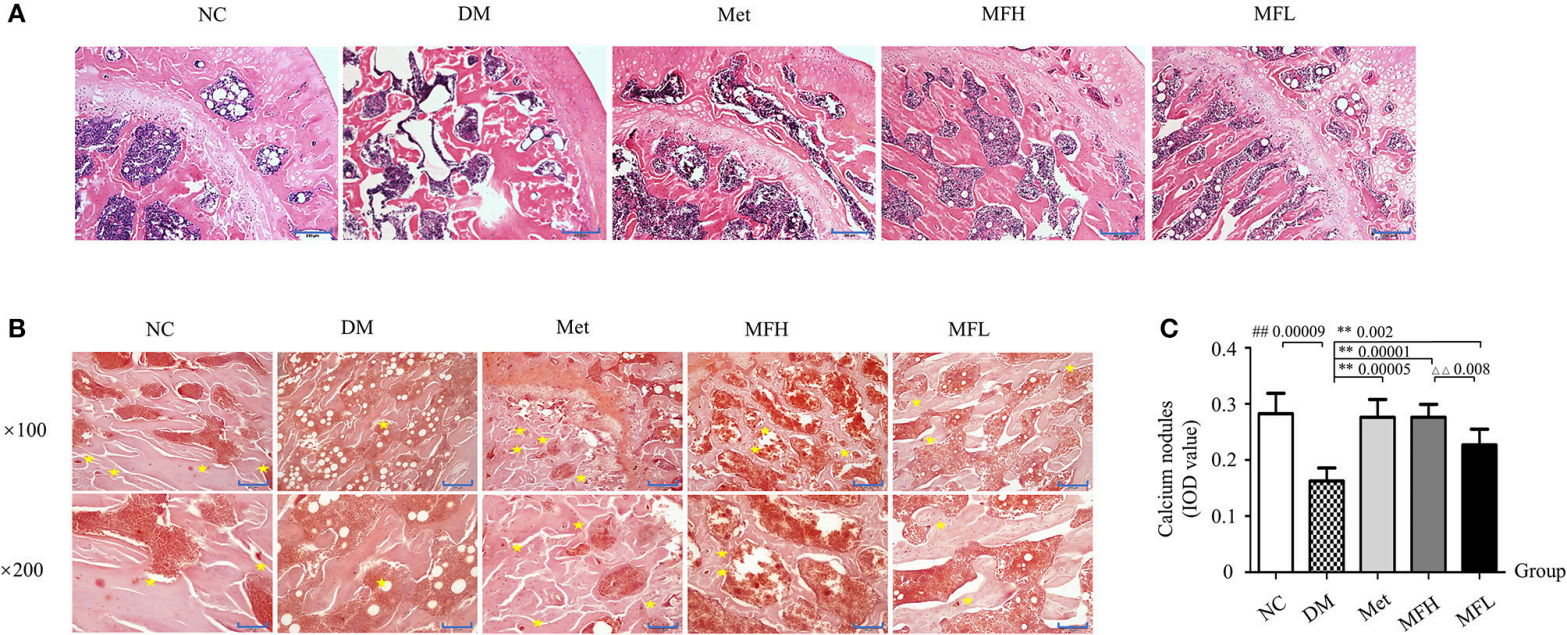
MF improved femoral microarchitecture in diabetic rats. **(A)** Shows the representative micro-images of H&E staining in the femurs of different groups. **(B,C)** Show the results of alizarin red S staining and their analyses in the different groups of rats ( × 100, Scale bar: 200 μm; × 200, Scale bar: 100 μm). Yellow star denotes calcified nodules. Data are presented as mean ± SD. *n* = 10. ^#^*p* < 0.05 or ^##^*p* < 0.01 compared with NC group, **p < 0.01 compared with DM group, ^ΔΔ^*p* < 0.01 compared with MFL group.

As shown in Figure 2A, H&E staining revealed a dense and regular meshwork of trabecular bone in rat femurs of the NC group. In contrast, the trabecular bones in rat femurs of the vehicle treated diabetic rats became thinner and irregular, and the trabecular bone reticulate structure was disorganized. Surprisingly, the trabecular bones turned thicker and organized in rat femurs of the Met, MFH, and MFL groups.

Alizarin red S reacts with calcium, which precipitated and formed calcified nodules during osteoblast differentiation and bone formation. As shown in Figures 2B,C, the distribution of calcified nodules was uneven and the areas of calcified nodules were markedly reduced in the rats of the DM group compared to those in the NC control (*p* < 0.01). Interestingly, administration of Met or MF aqueous extract (MFH and MFL) to diabetic rats for 12 weeks obviously improved the distribution and increased the areas of calcified nodules as compared to vehicle treated diabetic rats (*p* < 0.01). Additionally, MFH has better capacity in improving bone pathological disorders than MFL (*p* < 0.01).

### MF improved bone strength, biomechanical properties, and material properties in diabetic rats

**Figure 3:**
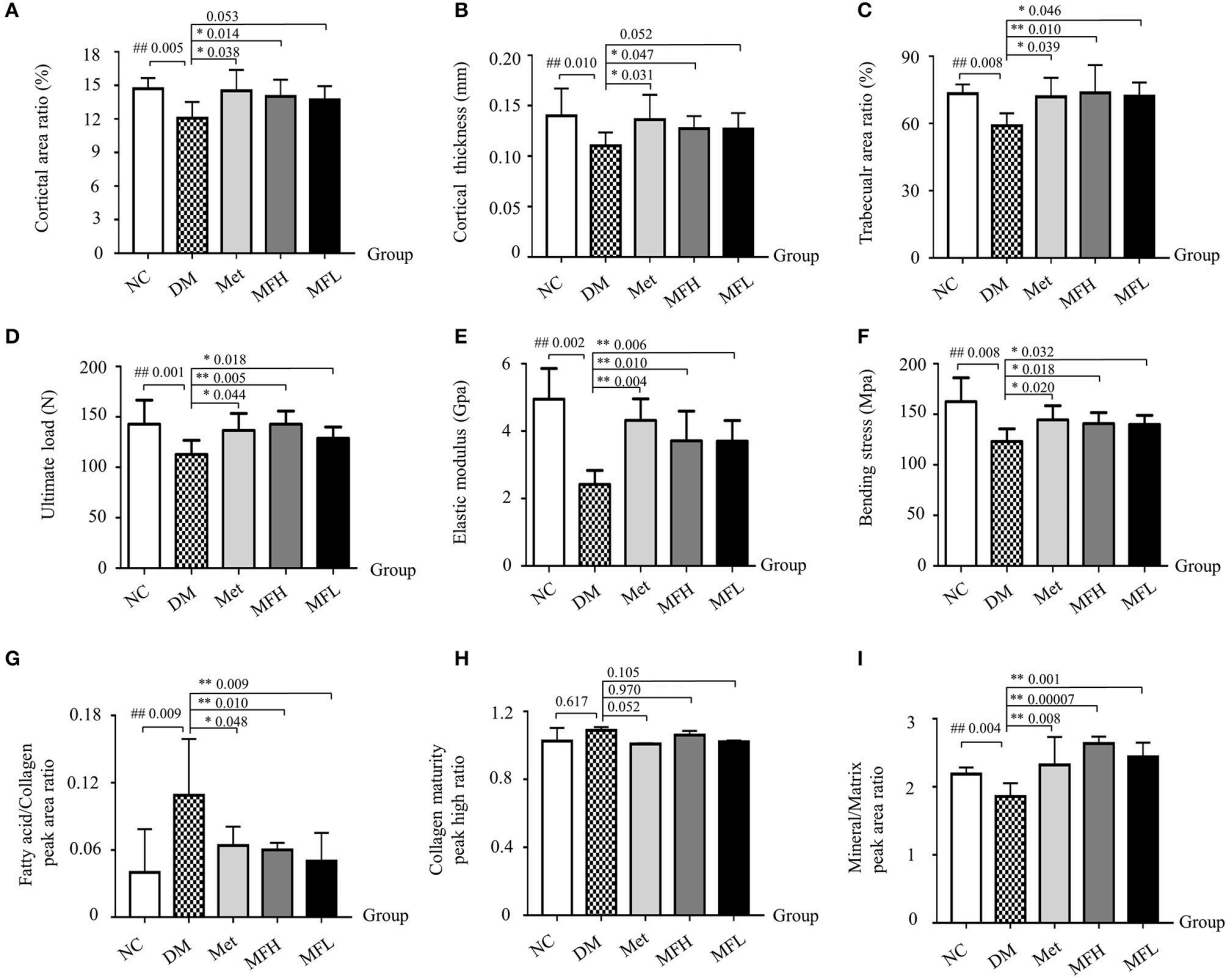
MF preserved bone strength, biomechanical, and material properties in the femurs of diabetic rats. **(A–C)** Show cortical area ratio, cortical thickness, and trabecular area ratio in different groups of rats determined by μCT Scanning. **(D–F)** Show the results of three-point bending examination of ultimate load, elastic modulus, and bending strength in different groups of rats. **(G–I)** Show the results of fatty acid/collagen, collagen maturity, and mineral/matrix detected by FTIR spectrum. Data are presented as mean ± SD. *n* = 9. ^#^*p* < 0.05 or ^##^*p* < 0.01 compared with NC group, *p < 0.05 or **p < 0.01 compared with DM group.

As shown in Figures 3A–C, the cortical area ratio, cortical thickness and trabecular area ratio were significantly reduced in rat femurs of the DM group when compared with those in the NC group (*p* < 0.01). Treatment of diabetic rats with Met or MFH aqueous extract obviously increased cortical area ratio, cortical thickness, and trabecular area ratio compared to the vehicle treated diabetic rats (*p* < 0.05). However, MFL treatment only increased trabecular area ratio, but did not exert effect on cortical area ratio and cortical thickness in the diabetic rats.

To further investigate the effect of MF aqueous extract on the bone biomechanical properties, the rat femurs were subjected to a three-point bending assay. As shown in Figures 3D–F, the ultimate load, bending strength and elastic modulus were significantly reduced in rat femurs of the DM group when compared with those in the NC group (*p* < 0.01). In contrast, treatment with Met or MF aqueous extract (MFH and MFL) to diabetic rats evidently increased the values of the ultimate load, bending strength, and elastic modulus in rat femurs compared to those of vehicle treated diabetic rats (*p* < 0.01 or 0.05).

Bone material properties, such as fatty acid/collagen, collagen maturity, and mineral/matrix were detected by FTIR spectrum. As shown in Figures 3G–I, the relative ratio of fatty acid/collagen was significantly increased in rat femurs of the DM group compared to that of the NC group (*p* < 0.01). Treatment of diabetic rats with Met or MF aqueous extract (MFH and MFL) significantly decreased the ratio of fatty acid to collagen compared to vehicle treated diabetic rats (*p* < 0.01 or 0.05). In addition, the relative ratio of mineral to matrix was markedly lower in vehicle treated diabetic rats than in NC controls (*p* < 0.01), and treatment with MF aqueous extract (MFH and MFL) or Met significantly reversed the ratio in diabetic rats (*p* < 0.01). Additionally, MFH has a good advantage over MFL in increasing the ratio of mineral to matrix than MFL (*p* < 0.05). However, there is no significant difference in collagen maturity of rat femurs between different groups (Figure 3H). These results indicate that MF aqueous extract has the ability of increasing bone strength and microstructure as well as improving bone material properties in diabetic rats.

### Effect of mf on igf-1, runx2, cathepsin k, opg, and rankl expressions in femurs and tibias of diabetic rats

**Figure 4:**
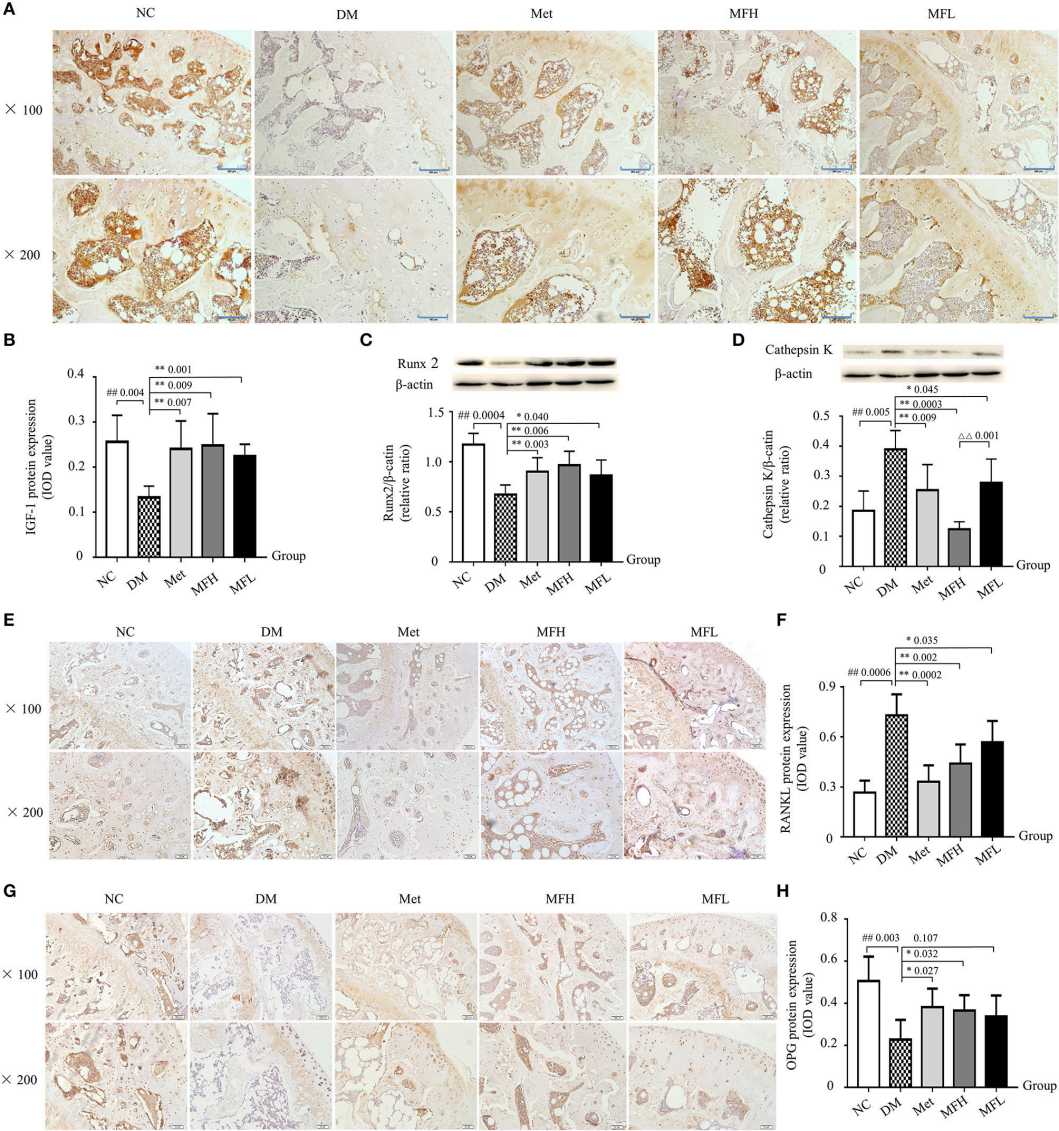
Treatment of diabetic rats with MF promoted bone formation and inhibited bone resorption in the tibias and femurs. The representative micro-images of IHC staining (sections were counterstained with hematoxylin; original magnification, × 100, Scale bar: 200 μm; × 200, Scale bar: 100 μm) and their analyses show the expressions of IGF-1 **(A,B)**, RANKL **(E,F)**, and OPG **(G,H)** in the femurs of different groups of rats. **(C,D)** Show the representative western blot images and their analyses of protein expressions of Runx2 and cathepsin K in the tibias of different groups of rats. Data are presented as mean ± SD. *n* = 10. The dark brown particles denote positive staining. IOD denotes integrated optical density of interested areas. ^#^*p* < 0.05 or ^##^*p* < 0.01 compared with NC group, *p < 0.05 or **p < 0.01 compared with DM group, ^ΔΔ^*p* < 0.01 compared with MFL group.

As shown in Figures 4A–C, the expressions of IGF-1 and Runx2 were decreased in rat femurs and tibias of the DM group compared with those of the NC group (*p* < 0.01). After treatment with MF aqueous extract (MFH and MFL) for 12 weeks, the expressions of IGF-1 and Runx2 were significantly increased in rat femurs of the DM group (*p* < 0.01 or 0.05).

Moreover, we evaluated the effect of MF on bone resorption. As shown in Figures 4D–F, the expressions of cathepsin K and RANKL were significantly increased in rat tibias of the DM group compared with that of the NC group (*p* < 0.01). Intriguingly, treatment with MF aqueous extract (MFH and MFL) or Met significantly decreased the expressions of cathepsin K and RANKL expression in the tibias of diabetic rats compared to vehicle treated diabetic control (*p* < 0.01 or 0.05). Additionally, MFH treatment showed stronger ability in reducing cathepsin K expression than MFL (*p* < 0.01). In contrast, treatment with MFH for 12 weeks significantly increased the OPG expression in the femurs of diabetic rats as compared to vehicle treated diabetic controls (Figures 4G,H; *p* < 0.05). However, MFL treatment did not exert significant effect on OPG expression in the femurs of diabetic rats. These results suggest that MF has the ability of promoting bone formation and suppressing bone resorption in the diabetic rats.

### Mf improved calcium homeostasis in diabetic rats

**Figure 5:**
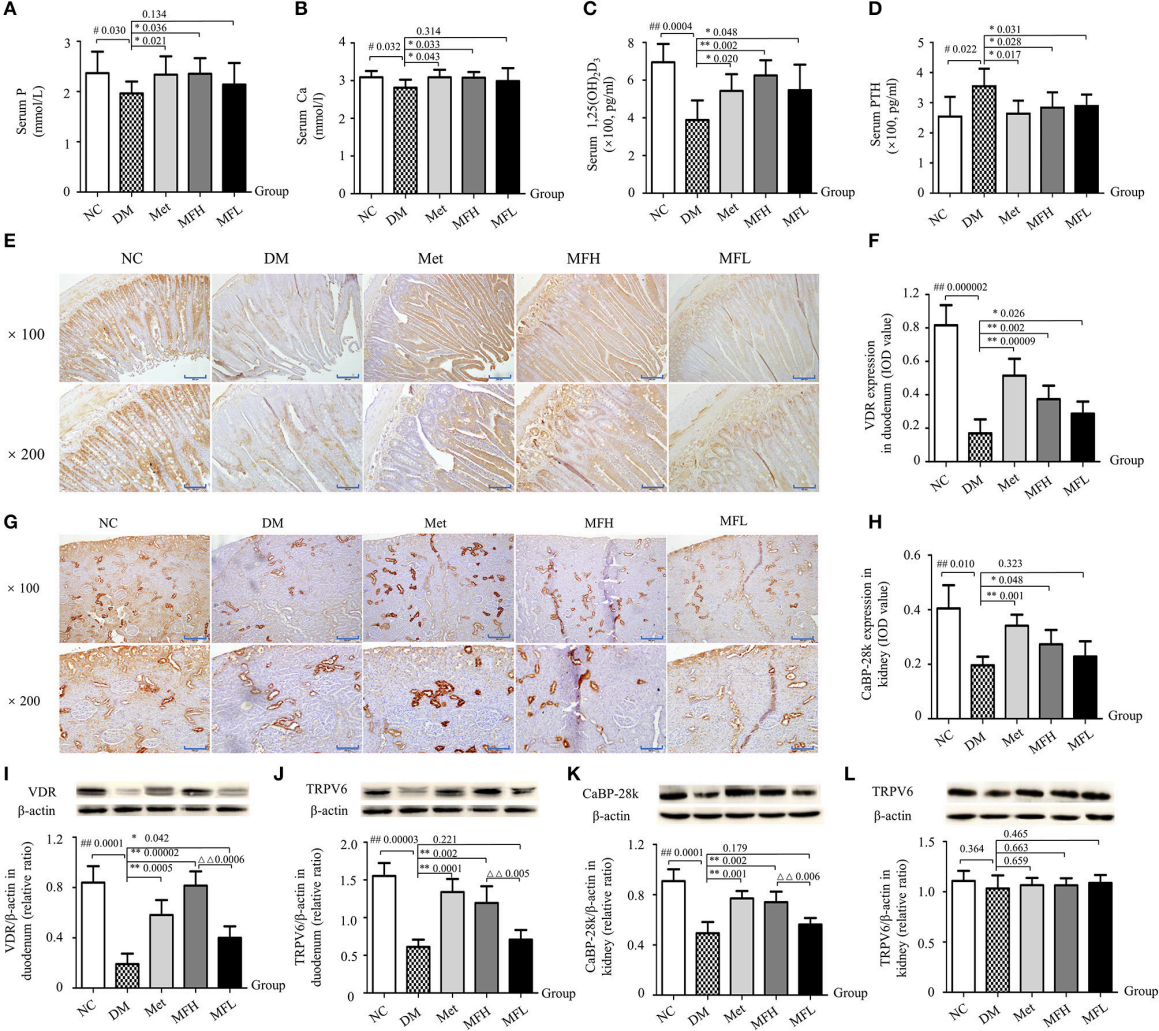
MF treatment improved calcium homeostasis through regulation of PTH/VDR/CaBP signaling in the duodenums and kidneys of diabetic rats. **(A–D)** Show serum levels of P, Ca, 1,25(OH)_2_D_3_ and PTH in the different groups of rats. **(E–H)** Show the representative micro-images of IHC staining (sections were counterstained with hematoxylin; original magnification, × 100, Scale bar: 200 μm; × 200, Scale bar: 100 μm) and their analyses of the levels of VDR in the duodenums and CaBP-28k in the kidneys of different groups of rats. **(I–L)** Show the representative images of western blot and their analyses of protein expressions of VDR and TRPV6 in the duodenums, and CaBP-28k and TRPV6 in the kidneys of different groups of rats. Data are presented as mean ± SD. *n* = 10. The dark brown particles denote positive staining. IOD denotes integrated optical density of interested areas. ^#^*p* < 0.05 or ^##^*p* < 0.01 compared with NC group, *p < 0.05 or **p < 0.01 compared with DM group, ^ΔΔ^*p* < 0.01 compared with MFL group.

Ca homeostasis *in vivo* is regulated by calciotropic hormones, including parathyroid hormone and 1,25(OH)_2_D_3_ (Dong et al., 2012). 1,25(OH)_2_D_3_, could stimulate intestinal Ca absorption and regulate bone metabolism by binding to VDR (Zhang et al., 2011, 2014). As shown in Figures 5A,B, the serum levels of Ca and P in the vehicle treated diabetic rats was lower than those in NC controls (*p* < 0.05). And Met and MFH treatments significantly increased serum levels of Ca and P in the diabetic rats (*p* < 0.05). In addition, compared with the rats in NC group, 1,25(OH)_2_D_3_ levels were decreased and PTH levels were increased in the serum of the DM group (Figures 5C,D; *p* < 0.01 or 0.05). Interestingly, treatments with Met, MFH and MFL for 12 weeks, the serum levels of 1,25(OH)_2_D_3_ and PTH were significantly reversed in the diabetic rats (*p* < 0.05).

To further explore the potential mechanism of MF aqueous extract on Ca homeostasis, the expressions of calcium transporting proteins were observed in rat duodenums and kidneys by western blot and IHC staining. As shown in Figures 5E,F,I,J, the expressions of VDR and TRPV6 were reduced in the duodenums of vehicle treated diabetic rats as compared to those of NC controls (*p* < 0.01). Interestingly, the downregulation of VDR and TRPV6 in the duodenums of diabetic rats were significantly inhibited upon Met and MFH treatment as compared to vehicle treated diabetic controls (*p* < 0.01 or 0.05). However, MFL treatment did not markedly affect TRPV6 in the duodenums of diabetic rats. In addition, the results from IHC staining and western blot (Figures 5G,H,K) showed that CaBP-28k expression was reduced in rat kidneys of the DM group as compared to those in the NC group (*p* < 0.01). Supplementation of Met or MFH aqueous extract to diabetic rats increased CaBP-28k in the kidneys when compared with vehicle-treated diabetic controls (*p* < 0.01 or 0.05). However, MFL treatment did not significantly increased CaBP-28k in the kidneys of diabetic rats. Moreover, MFH is more efficient in improving expression of VDR in the duodenums than MFL (*p* < 0.01). Surprisingly, hyperglycemia stimulation did not alter TRPV6 expression in rat kidneys (Figure 5L). And MF and Met treatments also did not affect TRPV6 expression in diabetic rat kidney (Figure 5L). These results suggest that MF may improve calcium metabolism through the regulation of PTH/VDR/CaBP signaling pathway.

### Mf ameliorated oxidative stress in diabetic rats

**Figure 6:**
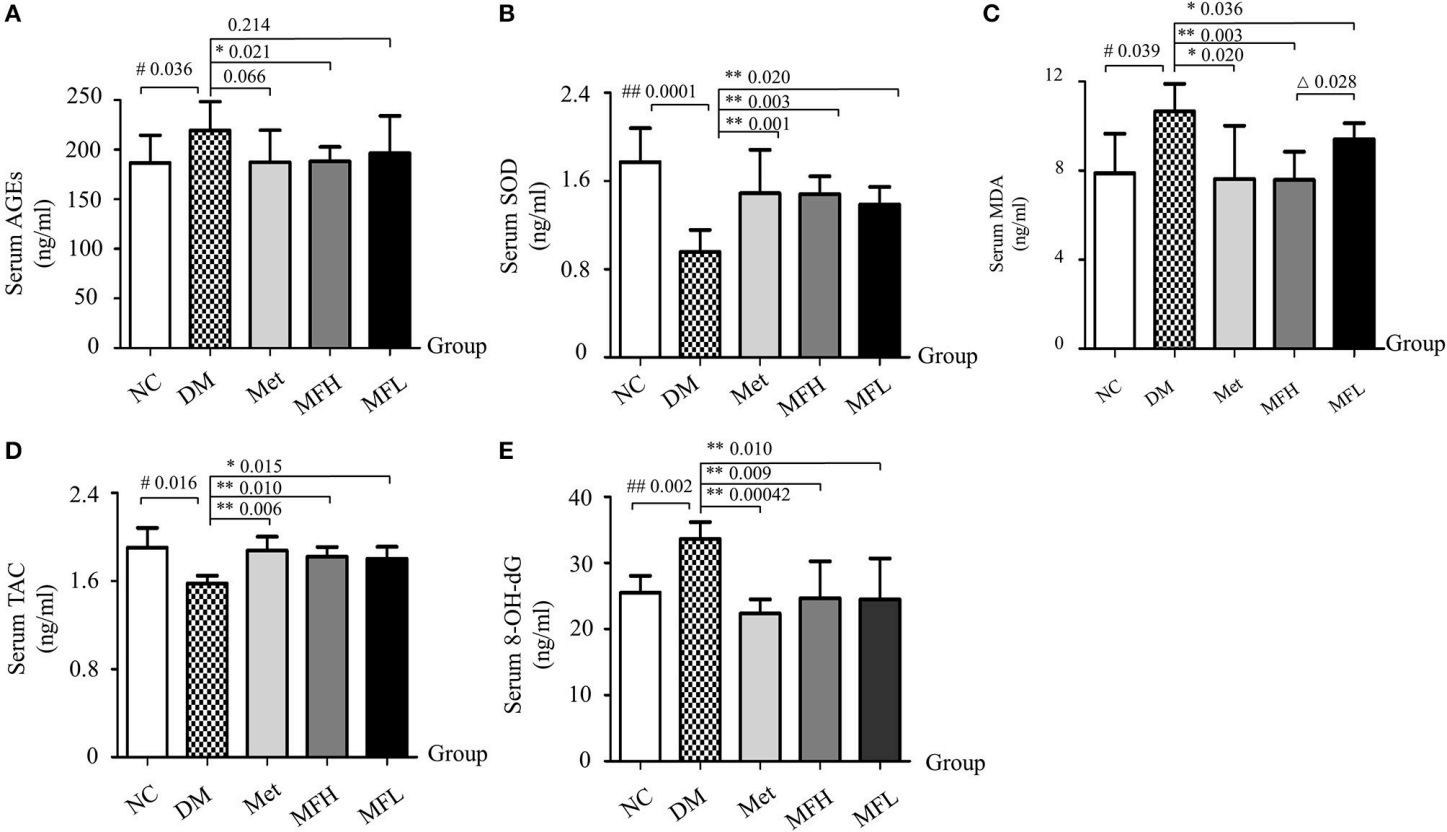
MF treatment restored serum redox markers in diabetic rats. **(A–E)** Show effect of MF on serum levels of AGEs, SOD, MDA, TAC, and 8-OH-dG in the different groups of rats. Serum samples from 12 rats per group were taken for each assay. Data are presented as mean ± SD. ^#^*p* < 0.05 or ^##^*p* < 0.01 compared with NC group, *p < 0.05 or **p < 0.01 compared with DM group, ^Δ^*p* < 0.05 compared with MFL group.

The disrupted redox homeostasis is one of main causes that compromised bone strength in diabetes (Napoli et al., 2017). Next, we examined the effect of MF on oxidative status in the diabetic rats. As shown in Figures 6A–E, the levels of AGEs, MDA, and 8-OH-dG were increased, and the levels of SOD and TAC were decreased in the vehicle treated diabetic rats, respectively, when compared with those of NC controls (*p* < 0.01 or 0.05). Interestingly, supplement of diabetic rats with Met and MF aqueous extract (MFH and MFL) attenuated the increased levels of MDA, and 8-OH-dG, and the decreased levels of SOD and TAC in the serum of the diabetic rats (*p* < 0.01 or 0.05) when compared with those of vehicle treated diabetic controls (*p* < 0.01 or 0.05). Moreover, MFH, rather than MFL, treatment significantly decreased AGEs levels in the serum of diabetic rats (Figure 6A). Additionally, MFH has better capacity in decreasing the MDA level in diabetic rats than MFL (*p* < 0.05).

**Figure 7:**
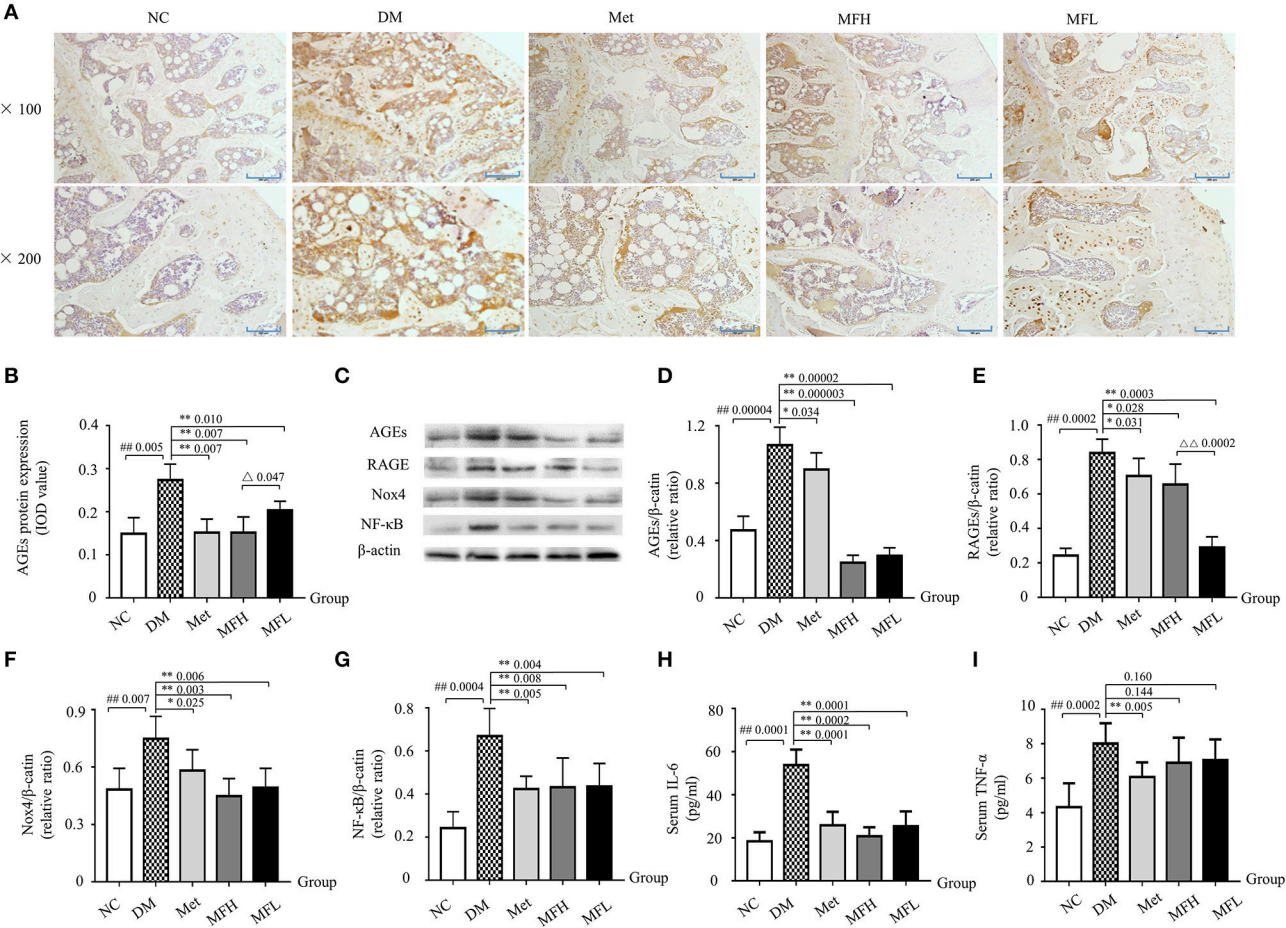
MF treatment improved bone quality through regulation of AGEs/RAGE/Nox4/NF-κB signaling in the tibias and femurs of diabetic rats. **(A,B)** Show the representative micro-images of IHC staining (sections were counterstained with hematoxylin; original magnification, × 100, Scale bar: 200 μm; × 200, Scale bar: 100 μm; *n* = 10) and their analyses of AGEs expressions in the femurs of different groups of rats. **(C–G)** Show the representative images of western blot and their analyses of protein expressions of AGEs, RAGE, Nox4, and NF-κB in the tibias of different groups of rats (*n* = 10). The dark brown particles denote positive staining. **(H,I)** Show the serum levels of IL-6 and TNF-α in the different groups of rats (*n* = 12). IOD denotes integrated optical density of the interested areas. Data are presented as mean ± SD. ^#^*p* < 0.05 or ^##^*p* < 0.01 compared with NC group, *p < 0.05 or **p < 0.01 compared with DM group, ^Δ^*p* < 0.05 or ^ΔΔ^*p* < 0.01 compared with MFL group. The expression of AGEs, RAGE, Nox4, and NF-κB were detected by stripping and redeveloping the same membrane with different primary antibodies. The amounts of protein in each lane was verified by redeveloping the membrane with β-actin.

The accumulation of AGEs may lead to activation of RAGE, which further enhanced ROS production and subsequent bone fragility in response to hyperglycemia (Napoli et al., 2017). As shown in Figures 7A–E, the results from IHC staining and western blot demonstrated that the expressions of AGEs and RAGE in the femurs and tibias were significantly increased in the vehicle treated diabetic rats when compared to the NC littermates (*p* < 0.01 or 0.05). Intriguingly, treatment with Met and MF aqueous extract (MFH and MFL) significantly reversed the above-mentioned biomarkers in diabetic rats (*p* < 0.01 or 0.05). And MFH is more efficient in reducing AGEs and RAGE expressions in diabetic rats than MFL (*p* < 0.01 or 0.05).

It is well-known that sustained hyperglycemia induces an upregulation of Nox (De Blasio et al., 2017), which further aggravates oxidative stress and subsequent bone resorption (Sankar et al., 2015). Next, we examined the alterations of Nox4 and NF-κB in the tibias of diabetic rats by western blotting. As shown in Figures 7C,F,G, the expressions of Nox4 and NF-κB were significantly increased in the tibias of vehicle treated diabetic rats when compared to those of the normal controls (*p* < 0.01). Accordingly, both Met and MF aqueous extract (MFH and MFL) treatment significantly inhibited the upregulation of Nox4 and NF-κB in diabetic rats when compared with vehicle treated controls (*p* < 0.01 or 0.05).

Enhanced expression of AGEs in diabetes not only provokes NF-κB activation, but also leads to an subsequent increase in IL-6 and TNF-α release (Zhou et al., 2016), which further exacerbates bone resorption (Blaschke et al., 2018). As anticipated (Figures 7H,I), the serum levels of IL-6 and TNF-α were significantly increased in the vehicle treated diabetic rats when compared with those in NC littermates (*p* < 0.01). And supplement of diabetic rats with Met and MF aqueous extract (MFH and MFL) alleviated the increased IL-6 levels in the diabetic rats (*p* < 0.01). However, MF treatment did not markedly affect serum TNF-α release in diabetic rats, but Met did. These observations suggest that MF may attenuate oxidative and inflammatory stress through downregulating the levels of AGEs, RAGE, Nox4, NF-κB, and IL-6 in diabetic rats.

## Discussion

**Figure 8:**
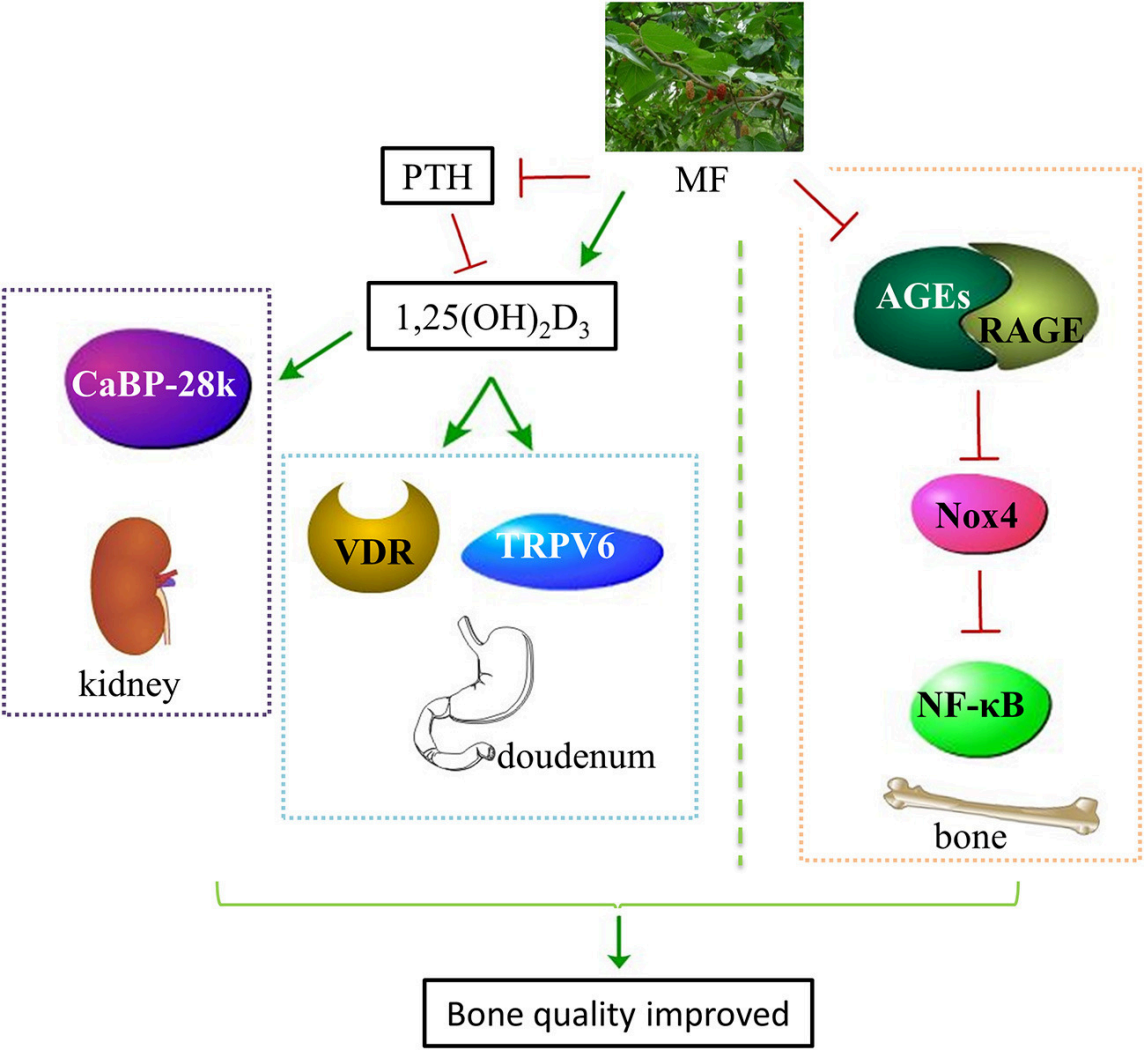
Schematic diagram illustrated the underlying mechanisms of MF aqueous extract in improving bone quality of diabetic rats. Administration of MF aqueous extract to diabetic rats improves calcium homeostasis through increasing the level of 1,25(OH)_2_D_3_ and decreasing the level of PTH as well as promoting VDR and TRPV6 expressions in the duodenum and CaBP-28k expression in the kidneys. Meanwhile, MF also rebuilds redox balance through reducing the expressions of AGEs, RAGE, Nox4, and NF-κB in the femurs and tibias of diabetic rats. MF, Mori Folium; 1,25(OH)2D3, 1,25-dihydroxyvitamin D3; PTH, parathormone; VDR, vitamin D receptor; TRPV6, transient receptor potential V6; CaBP-28k, calcium-binding protein-28k; AGEs, advanced glycation end products; RAGE, receptor of advanced glycation end products; Nox4, NADPH oxidoreductase 4; NF-κB, nuclear factor kappa-B.

In the present study, we first revealed that administration with aqueous extract of MF to diabetic rats reduced FBG, improved glucose tolerance, and increased the levels of OC, IGF-1, P, Ca, 1,25(OH)_2_D_3_, SOD, and TAC as well as decreased the levels of TRAP, PTH, AGEs, IL-6, MDA, and 8-OH-dG in the serum. Secondly, MF treatment improved bone microstructure and material properties, and increased bone strength in the femurs of diabetic rats. Thirdly, we demonstrated that MF treatment also increased the expressions of IGF-1, OPG, and Runx2, decreased the expressions of cathepsin K and RANKL as well as downregulated the levels of AGEs, RAGE, Nox4, and NF-κB in the femurs and tibias of diabetic rats. Last but not least, we also found that MF also upregulated the expressions of CaBP-28K, TRPV6, and VDR in the kidneys or duodenums of diabetic rats (Figure 8).

Our current study discovered that MF could reverse deterioration of bone microstructure and reduction of bone strength as well as improve bone material properties in the diabetic rats induced by HSD-HFD and STZ. Previously, we have demonstrated that MF exerted hypoglycemic effect in diabetic rats and mice (Jiao et al., 2017; Riche et al., 2017). Since diabetes always induces higher risk of bone fracture (Al-Hariri, 2016), we then observed the effect of MF on bone alterations in the development of diabetes. We found that MF treatment increased thickness and improved distribution of femoral trabecular bone in the diabetic rats. Our findings also demonstrated that administration with MF for 12 weeks to diabetic rats decreased the relative ratio of fatty acid to collagen as well as increased the relative ratio of mineral to matrix. It is known that the disturbances in bone microstructure and material properties may compromise bone strength and increase bone fragility (Acevedo et al., 2018). Accordingly, we demonstrated that MF treatment increased the capacity of ultimate load, resist bending, and elastic tension. This is in line with the previous findings that *M. alba* combined with *P. odoratum* could increase cortical bone thickness and osteoblast numbers, and reduce osteoclast numbers in OVX induced osteoporotic rats (Sungkamanee et al., 2014). Together, the current findings suggest that MF has the ability of improving bone quality in diabetic rats.

The present study demonstrated that the expressions of IGF-1, OPG, and Runx2 were upregulated and the expressions of cathepsin K and RANKL was downregulated in the femurs and tibias of diabetic rats following supplement of MF, which was consistent with our previous findings (Guo et al., 2016). We also found that MF treatment suppressed IL-6 release as well as NF-κB expression in the diabetic rats. Pezhman et al. demonstrated that diabetes may affect bone remodeling and increase bone loss through increased RANKL/OPG ratio (Pezhman et al., 2018). Additionally, the decreased serum IGF-1 level in diabetic patients may contribute to the development of osteopenia (Teppala and Shankar, 2010). In contrast, an increase in IGF-1 expression promotes osteoblastogenesis and inhibits osteoclastogenesis (Guerra-Menéndez et al., 2013). Moreover, the increased cathepsin K expression in diabetic rats and mice (Hie et al., 2007; El-Maraghy and Mehana, 2015) promotes bone resorption and accordingly compromises bone quality. Meanwhile, upregulation of cathepsin K prevents MSCs differentiation into osteoblasts (Nuttall et al., 2014). In addition, the decreased expression of Runx2 in diabetes further inhibits osteoblastogenesis (Qian et al., 2016). Besides, diabetes could aggravate bone loss through promoting NF-κB activation and IL-6 and TNF-α release (Zhen et al., 2015; Zhou et al., 2016). Therefore, it is conceivable that MF may promote bone formation through upregulation of IGF-1, OPG, and Runx2 as well as downregulation of cathepsin K and RANKL expressions in diabetic rats.

In the current study, we first demonstrated that MF increased Ca level and decreased PTH level in the serum of the diabetic rats. It is accepted that high level of PTH is negatively correlated with BMDs and 1,25(OH)_2_D_3_ as well as positively correlated with high risk of bone fracture (Wang et al., 2018). In addition, low level of 1,25(OH)_2_D_3_ may be linked to development of type 2 diabetes. Increased level of 1,25(OH)_2_D_3_ favors glucose homeostasis and bone formation. Interestingly, we currently found that MF could increase calcium nodules in rat femurs of the diabetic rats by alizarin red S staining. Additionally, MF in combination with Konjac Glucomannan or *P. odoratum* could increase serum Ca level in low calcium diet feeding (Yan, 2017) and osteoporotic rats (Sungkamanee et al., 2014). Rutin, one of main constituents in MF aqueous extract, has been demonstrated to prevent osteoclastogenesis through inhibition of ROS production in the presence of 1,25(OH)_2_D_3_ (Rassi et al., 2005; Kanikarla-Marie and Jain, 2016). Therefore, it is reasonable to deduce that MF exhibits bone protective effect partly through improving calcium homeostasis by regulation of PTH and 1,25(OH)_2_D_3_ level in diabetic rats.

In the present study, we demonstrated that MF increased calcium absorption in the intestine, and reduced calcium excretion and increased calcium reabsorption in the kidneys by increasing the expressions of TRPV6, CaBP-28k, and VDR. The low level of VDR potentiates calcium excretion in the duodenums of diabetic rats (Sha et al., 2017). In addition, the decreased TRPV6 and CaBP-28k expression in the duodenums and kidneys further resulted in calcium wasting and resultant bone loss (Zhang et al., 2008; Feng et al., 2014). Moreover, 1,25(OH)_2_D_3_ in combination with vitamin K2 has been proved to enhance calcium deposits in the osteoblasts of diabetic mice (Poon et al., 2015). In addition, treatment with 1,25(OH)_2_D_3_ reduces the incidence of diabetes and its associated bone loss (Del Pino-Montes et al., 2004). Therefore, MF may accomplish bone protective effect through the regulation of calcium metabolism in diabetic rats, which contributes to preventing the progress of diabetes.

Given that the involvement of oxidative stress in diabetic osteoporosis (Cheng et al., 2018) and the anti-oxidant ability of MF (Kim et al., 2014), AGEs, RAGE, Nox4, and NF-κB in the femurs and tibias of diabetic rats were determined. The results turned out that STZ plus HSD-HFD significantly disrupted the redox balance as evidenced by an increase in serum levels of oxidant markers (AGEs, MDA, and 8-OH-dG), and a decrease in serum levels of anti-oxidant markers (SOD and TAC), as well as an increase in the expressions of AGEs, RAGE, Nox4, and NF-κB in the femurs and tibias of diabetic rats. MF aqueous extract treatment reversed the alterations of redox status in the serum, femurs, and tibias of diabetic rats. Moreover, we found that the serum levels of above-mentioned redox markers and the expressions of AGEs, RAGE, Nox4, and NF-κB in the femurs and tibias of MFH group were almost restored to the initial levels in the normal group. In addition, MF has been proved to suppress IL-1β induced activation of NF-κB in SW1353 human chondrocytes. Furthermore, chlorogenic acid (Bagdas et al., 2015) and kaempferol (Pang et al., 2006), two of the ingredients in MF, have been reported to exhibit anti-oxidant activity in diabetic rats or RAW264.7 cells. Collectively, these results suggested that MF aqueous extract may improve bone quality through the regulation of redox homeostasis in diabetic rats.

In conclusion, we demonstrate that MF aqueous extract exerts bone protective activity through an improvement of calcium homeostasis via regulating the PTH/VDR/CaBP signaling, and an elimination of oxidative stress via regulating the AGEs/RAGE/Nox4/NF-κB signaling in diabetic rats. These results may suggest the potential of MF in preventing the development of diabetic osteoporosis. However, further studies are needed to elucidate the active constituents in MF aqueous extract that assuming the bone protective effects, which will contribute to identify the antagonists for diabetic osteoporosis.

## Author contributions

CL, RZ, and DZ conceived and designed study; CL, RZ, HL, LL, BC, QJ, LW, RM, ST, and MW conducted study; MF, JN, AO, and SG analyzed data; CL, RZ, MF, and DZ wrote the paper. BZ and DZ have primary responsibilities for final content. All authors have read and approved the final manuscript.

### Conflict of interest statement

The authors declare that the research was conducted in the absence of any commercial or financial relationships that could be construed as a potential conflict of interest.

## Supplementary material

Supplementary Figure 1 Characterization and Identification of the main ingredients in the aqueous extract of *Mori Folium* (MF). **(A)** HPLC-MS chromatogram of MF. The peaks and chemical structures **(B)** were identified in chromatogram as followings: 1. p-Coumaric acid; 2. Chlorogenic acid; 3. Quercetin 3,7-*O*-diglucoside; 4. Kaempferol-3,7-O- diglucoside; 5. Isoquercitrin; 6. Astragalin; 7. Isochlorogenic acid C; 8. Rutin; 9. Hyperoside.
