# Supplementary figures and images for: Aqueous Extract of Mori Folium Exerts Bone Protective Effect Through Regulation of Calcium and Redox Homeostasis via PTH/VDR/CaBP and AGEs/RAGE/Nox4/NF-κB Signaling in Diabetic Rats
Source: Front Pharmacol. 2018 Nov 6;9:1239. doi: 10.3389/fphar.2018.01239 (PMC6233025; doi:10.3389/fphar.2018.01239)

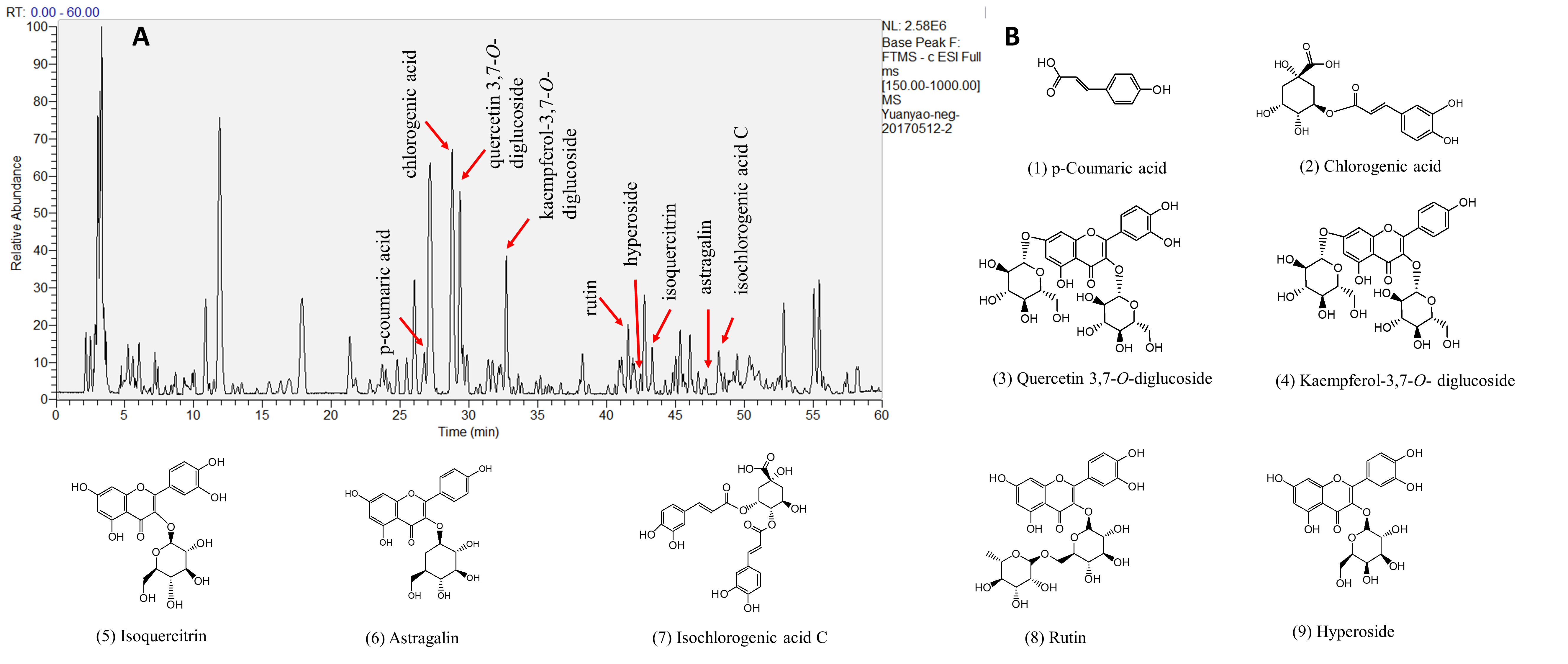

Supplement: Supplementary Figure 1 — Characterization and Identification of the main ingredients in the aqueous extract of Mori Folium (MF). (A) HPLC-MS chromatogram of MF. The peaks and chemical structures (B) were identified in chromatogram as followings: 1. p-Coumaric acid; 2. Chlorogenic acid; 3. Quercetin 3,7-O-diglucoside; 4. Kaempferol-3,7-O- diglucoside; 5. Isoquercitrin; 6. Astragalin; 7. Isochlorogenic acid C; 8. Rutin; 9. Hyperoside. [file Image_1.TIF]
